# Time to appreciate Avicenna’s knowledge of rabies

**Published:** 2017

**Authors:** Nader Aghakhani, Mehdi azami, Mohsen Ghomashlooyan, Alireza Nikoonejad

**Affiliations:** 1 Department of Medical Surgical Nursing, Faculty of Nursing and Midwifery, Urmia University of Medical Sciences, Urmia, Iran.; 2 Skin Diseases and Leishmaniasis Research Center, Isfahan University of Medical Sciences, Isfahan, Iran.; 3 Department of Parasitology, Shahrekord Branch, Islamic Azad University, Shahrekord, Iran.; 4 Department of Parasitology and Mycology, School of Medicine, Isfahan University of Medical Science, Isfahan, Iran.; 5 Department of Infectious Disease, Taleghani Hospital, Urmia University of Medical Sciences, Urmia, Iran.

**Keywords:** Rabies, Avicenna, Traditional medicine, Treatment

The treatment of infectious diseases determines its history back to the research of many scientists, such as Persian scholars. The outstanding Persian scientist Avicenna (980-1037 AD) who is remembered for his contributions to the science of medicine was a master in curing many diseases like rabies. 

It is possible to analyze Avicenna’s opinions about rabies and his contributions to this problem of medicine through a study of his famous and great medical manuscript Al-Qanun-phi-al-Tibb (The Canon of Medicine) which is one of the most important textbooks ever written on many medical issues such as rabies (1). 

Rabies is an acute, progressive, and fatal anthropozoonotic infection that affect the central nervous system and is estimated that approximately 100,000 people die from it each year, although the true number is much higher because of underreporting (2, 3). 

In Iranian traditional medicine, the word rabies means madness that is transmitted through a close contact with infected saliva via bites or scratches (4). Iranian physicians have written much and introduced their innovations in their works now and before (5).

 Avicenna suggested that the imbalance of the four humors causes different diseases like rabies. Based on his view, therapeutic interventions should be done to maintain the balance of humors in the body. He recommended cupping and sucking the site of the bite. It is necessary to evaluate the manifestations of rabies in dogs which are responsible for the bite. In writing his famous book, he gathered major medical information available from some of his predecessors, such as Rhazes (865–925), who described symptoms of rabies and wrote about them, and combined them with his clinical experiences (6, 7).

In summary, Avicenna described the symptoms of rabies in this book mentioned that it was caused by a toxic substance in the saliva of a rabid animal and stated that the disease will have a better prognosis if the bite site is bleeding and incurable unless the patient was admitted before hydrophobia, physicians must avoid suturing of the bite wound and leave sutures open to prevent it from healing if it was sutured by other physicians (4).

In conclusion, our medieval Persian scientists like Avicenna had discussed about rabies and he provided a complete description of the signs, prognosis and treatment of the disease. Therefore, we should be grateful to Avicenna for his significant role in the medicine during his own time. Understanding his knowledge about rabies can help us in comprehend understanding the progress of medicine and the views about the infectious diseases such as rabies in medieval times.
